# Re-Evaluate the Effect of Hyperbaric Oxygen Therapy in Cancer - A Preclinical Therapeutic Small Animal Model Study

**DOI:** 10.1371/journal.pone.0048432

**Published:** 2012-11-07

**Authors:** Sneha Pande, Amit Sengupta, Anurag Srivastava, Rajiv P. Gude, Arvind Ingle

**Affiliations:** 1 Bioengineering Laboratory, Advanced Centre for Treatment, Research & Education in Cancer, TATA Memorial Centre, New Mumbai, Maharashtra, India; 2 Department of Cancer Surgery, All India Institute of Medical Science, New Delhi, India; 3 Gude Laboratory, Advanced Centre for Treatment, Research and Education in Cancer, TATA Memorial Centre, New Mumbai, Maharashtra, India; 4 Laboratory Animal Facilities, Advanced Centre for Treatment, Research and Education in Cancer, TATA Memorial Centre, New Mumbai, Maharashtra, India; University of Tennessee, United States of America

## Abstract

Tumor hypoxia is a known driver of angiogenesis that also facilitates tumor growth. Moreover, poorly oxygenated central tumor area remains relatively radio or chemo resistant. HBO therapy is known to elevate the levels of dissolved oxygen and eliminates tumor hypoxia. It has been one of the modalities in cancer treatment; therefore its optimization is important. In this experimental study, no cancer enhancing effect was seen during the course of HBO therapy; however, post therapy there was an accelerated growth and progression of tumor. HBO treated mice lived shorter and the response to therapy was dose & tumor volume dependent. HBO therapy probably exert its effect on the cancer proliferating cells through multiple pathways such as increased DNA damage, apoptosis & geno-toxicity leading to slow cancer progression while post therapy tumorigenic effect could be due to impaired DNA repair mechanism, mutagenic effect & aneuploidy as well as altered blood supply & nutrients. Tumor growth reached plateau with time and this finding validated theoretical model predicting tumor reaching an asymptotic limit. While, marked asymmetry observed in tumor volume progression or cancer cell proliferation rate in each of the experimental C3H mouse suggested a need for an alternate small animal pre-clinical cancer therapeutic model.

## Introduction

Intense cancer research is underway to discover possible therapies but no major breakthrough appears to be in sight in terms of its cure when diagnosed late. Recently, there has been much interest to optimize oxygen therapy in cancer. However, over the years, oxygen therapy and in particular hyperbaric oxygen therapy (HBOT) is being used routinely to treat various conditions like radiation injury [1], non- healing traumatic [2], ischemic, diabetic [3] or radiation induced wounds, venous or arterial ulcers, burns, pressure sores [4] ^,^ bone infections, gangrene, air or gas embolism, CO or smoke poisoning, decompression sickness etc with success rate varying from 60% to 90%. HBOT also helped in recovery from sudden hearing loss with no apparent cause; it has also been tried in neuro-rehabilitation post head injury, stroke, cerebral palsy, Bell’s palsy or spinal injury with success rate varying from 17% to 54% [5]_._ HBOT prevents the death of the dormant brain cells in the oxygen deficient regions and revive them. It involved the administration of pure oxygen at pressure higher than the normal atmospheric pressure. Even though it is used as an adjuvant to chemotherapy and radiotherapy [6], [7] its effect on malignancy remains uncertain [8]. Though there is some evidence that HBO improves local tumor control mostly in head & neck and uterine cervix and not in other anatomical sites but these benefits may only occur with unusual fractionation schemes and associated significant adverse effects including oxygen toxic seizures and severe tissue radiation injury [9]. Hyperbaric oxygen is known to increase radiation & chemo induced cell kill, cell damage by free radicals (ROS), reduced recurrence due to suppressed cancer stem cells and compressed veins and lymphatic’s during surgery. It exerts its effect on the tumor microenvironment through various mechanisms. One of the mechanisms is by increasing the oxygen diffusion to the tissues by raising dissolved oxygen level & reactive oxygen species in plasma. The basis of modulating pressure and oxygen concentration is to regulate cellular metabolism or tumor microenvironment. High external pressure exerted on the arterioles or capillaries results in reduction in tumor microcirculation, compromising oxygen perfusion as well as glucose & micronutrient supply to the proliferating cell [10], [11]. The net effect on the tissue oxygen level during HBO therapy remains positive thus eliminating tumor hypoxia. Theoretical model and physiological fluid dynamic studies have also documented alteration in blood flow volume and tissue perfusion due to change in the extra-luminal vascular pressure [12], [13]. Hypoxia drives angiogenesis and facilitates tumor growth [10], [14]. At molecular or cellular level, reduced blood flow & micronutrient may inhibit cell proliferation and tumor growth. While at the same time, high level of free radicals generated during HBO therapy further reduces tumor growth and progression due to enhanced DNA damage & apoptosis [15]-[22]. On the contrary, some of the studies showed HBO to have cancer enhancing effects by accelerating tumor cells proliferation [8], [23]. HBO may mobilize stem/progenitor cells by stimulating NO synthesis [24], [25]. Thus, we thought of revisiting the controversy and accordingly designed this prospective experimental physiological study to redefine the role of HBO in conventional mice model. This study has a dual purpose i.e., validate known reported findings of other co-workers and find out usefulness of pre clinical small animal cancer therapeutic model used over the years by various investigators. Developing appropriate small animal pre-clinical cancer therapeutic experimental model is an important method of enquiry to assess various newer or adjuvant cancer treatment modality. The C3H/HeJ mouse mammary tumor system is one of the most extensively studied model systems available as of now yet problem continues with the failure to reproduce results obtained from such model in human being [26]. Genetically engineered mice have also been used as pre clinical cancer therapeutic model [15], [26]-[30]. Based upon the preliminary results obtained in our study, we tried to explain underlying pathophysiological mechanisms that may be linked to HBO therapy in terms of its dual effect i.e., tumor growth delay during therapy and tumor growth enhancement post therapy. Though our experimental results validated a well established theoretical model on cancer cell proliferation rate but we suggested a need for an alternative approach in pre therapeutic cancer modeling so that the results can be reproduced in human being.

## Materials and Methods

### Experimental Model

**Figure 1:**
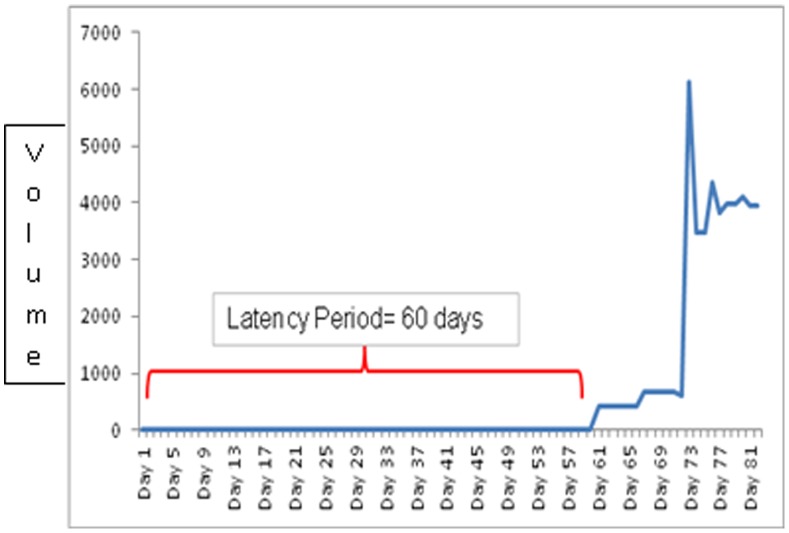
Graphical representation of latency period for tumor appearance.

**Figure 2:**
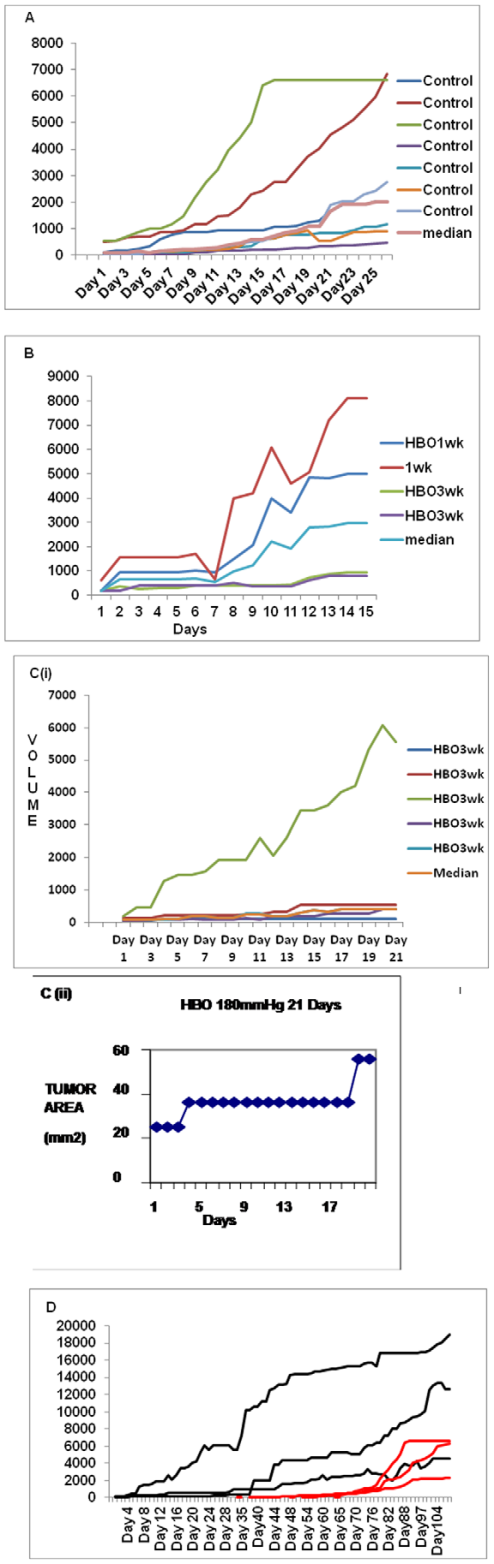
(A) Graphical representation of control mice (30 days follow up); (Anisometric growth in each mouse). (**B)** Graphical representation of C3H mice treated with HBO at 1.1 bar (1 week Vs 3 weeks: Growth suppression during therapy). (**C)** Graphical representation of HBO treated mice at 1.2 bar (during therapy). **(C [ii])** Growth retardation during HBO therapy (single plot). (**D)** Comparative trend of tumor progression of control and HBO treated C3H mice. (Black for HBO; Red for control).

**Figure 3:**
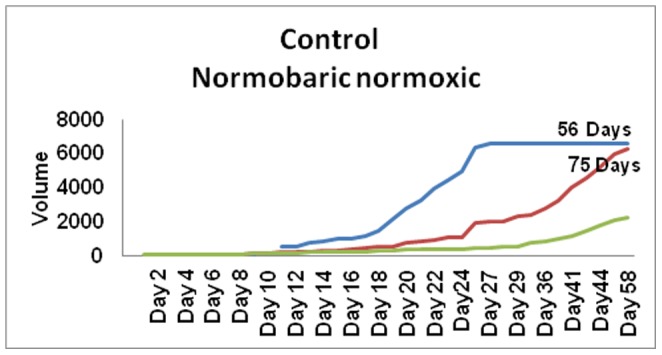
Growth pattern w.r.t the initial tumor volume.

Study was carried out in Laboratory Animal Facility (ACTREC) in strict accordance with the recommendations of the Committee for the Purpose of Control and Supervision of Experiments on Animals (CPCSEA), Ministry of Environment and Forests, Govt. of India. The protocol was approved by the Institutional Animal Ethics Committee (IAEC) of Tata Memorial Centre-Advanced Center for Treatment Research and Education in Cancer (Proposal No. 19/2010). All efforts were made to minimize suffering during the study. An inbred C3H strain with agouti coat color was used for this study. C3H strain has high incidence (70%) of mammary tumors and does not carry mouse mammary tumor virus (MMTV). It is now among the most widely used of all mouse strains. Homogeneity is the critical/fundamental point to be maintained in animal experimentation. However in case of mice, it is quite straightforward to develop genetically identical mice by inbreeding. These genetically identical mice can be used to replicate and compare experiments over time. Ik Soo Kim *et al*, 2010 explained about the mouse models for breast cancer metastasis. Emerging data have suggested that animal models are a good system to investigate this communication. Therefore, studies with mouse models have been developed as a systemic approach to understand breast cancer metastasis. Six to eight week old inbred C3H (Agouti) female mice were housed in cages with access to food and water ad *libitum* and exposed to 12 hr light dark cycle. The breeding system adopted in our animal facilities was 1∶1 brother sister mating till 20 generation to achieve isogenicity. The isogenicity has been further verified by regular quality control monitoring with biochemical markers (Hbb, Car-2, Idh, Mod), skin grafting, molecular markers (microsatellite) apart from regular health monitoring. A tumor cell suspension was prepared from aseptically excised spontaneous mammary tumor. Mammary tumors were induced using 0.1 ml subcutaneous injection of cell suspension (2.5×10^6^ cells/ml) in mammary fat pad. Daily inspection of the induced mice was done for food, water, bedding, comfort and appearance of the tumor nodule under all aseptic precaution by the same research fellow to minimize the error and reduce inter personal variation. The tumor measurements were noted from the day the nodule was palpated. The mice were subjected to experimental intervention from the time the nodule size measured between 5–10 mm in diameter. Macroscopic tumors were evident by day 60 (median value/mean value) following tumor induction – latent period (Figure 1). The growth pattern was shown in Figure 2 (A, B, C, D) & 3.

### HBO Protocol

Animals were exposed to Hyperbaric oxygen therapy at 1.1 (N = 10) and 1.2 bar (N = 5) for 2 hours in specifically designed Hyperbaric Chamber in our facility (ACTREC). HBO therapy was administered daily (1.1 b for 7 days in 2 mice), (1.1 b for 21 days in 8 mice), (1.2 b for 21 days in 5 mice).They were further followed up for tumor growth, body weight, survival period and metastasis. After death tissues (tumor, lung, brain, liver and heart) were collected for histological analysis. The detailed distribution is given in Table 1.

**Table 1:** Distribution of C3H mice subjected to HBO therapy showing survival duration & metastasis.

Study Groups	N	Tumor cell inoculation	Metastasis	Survival Range(Days)	Median Survival Value
Control	N = 7	2.5×10^6^	Not seen	52–143	104 days**
HBO^a^	N = 2*+8#	2.5×10^6^	Observed	86–95	91 days
HBO^b^	N = 5#	2.5×10^6^	Not seen	86–95	91 days

a Mice were subjected to HBO therapy at 1.1 bar for 120 minutes.

b Mice were subjected to HBO therapy at 1.2 bar for 120 minutes.

* Mice were treated with HBO therapy for 1 week.

#Mice were treated with HBO therapy for 3 weeks.

** P = 0.731 (Not significant)-Control Vs HBO.

### Measurements of Tumor Growth

Tumor size was measured using calipers at first tumor nodule appearance, considered as day 1 followed with daily measurements till the end point of study i.e., natural demise or when decision was taken by the investigator to euthanize mice due to heavy tumor burden with associated morbidity. Tumor growth was calculated according to the formula: V =  L×(W) ^2^/2 and by 4πr^3^ (plot is same). Daily weight measurements of the animal were done until they survived. Statistical analysis was carried out by finding out the median and mean values of the tumor volume over the period of time and the survival duration. The level of significance was taken as *P<*0.05, by Mann Whitney test and results are expressed as median & means±2SE (25th to 75^th^ percentile).

### Developing a Theoretical Predictive Model and Model Validation

**Figure 4:**
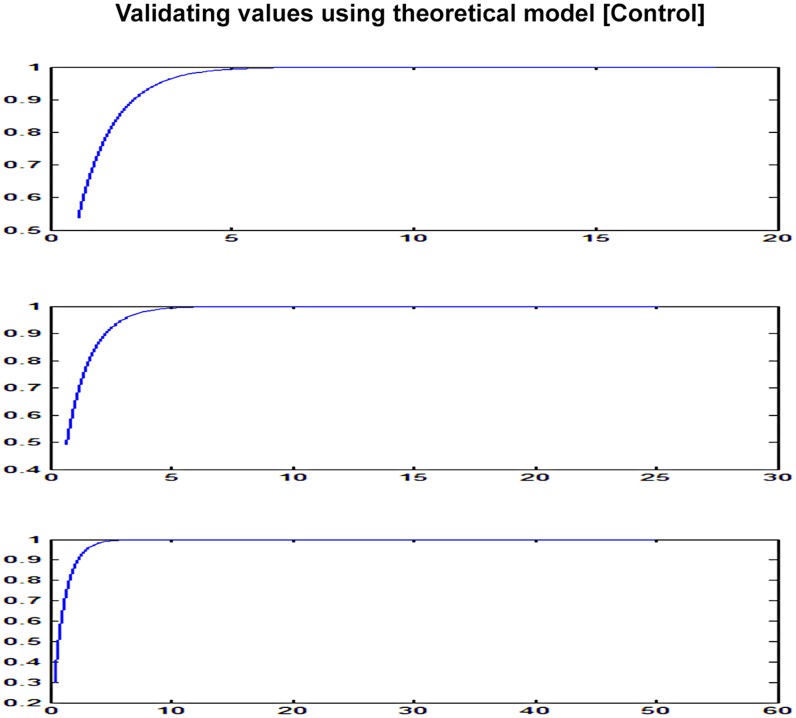
Validating theoretical model-prediction (control) - initial rapid growth.

**Figure 5:**
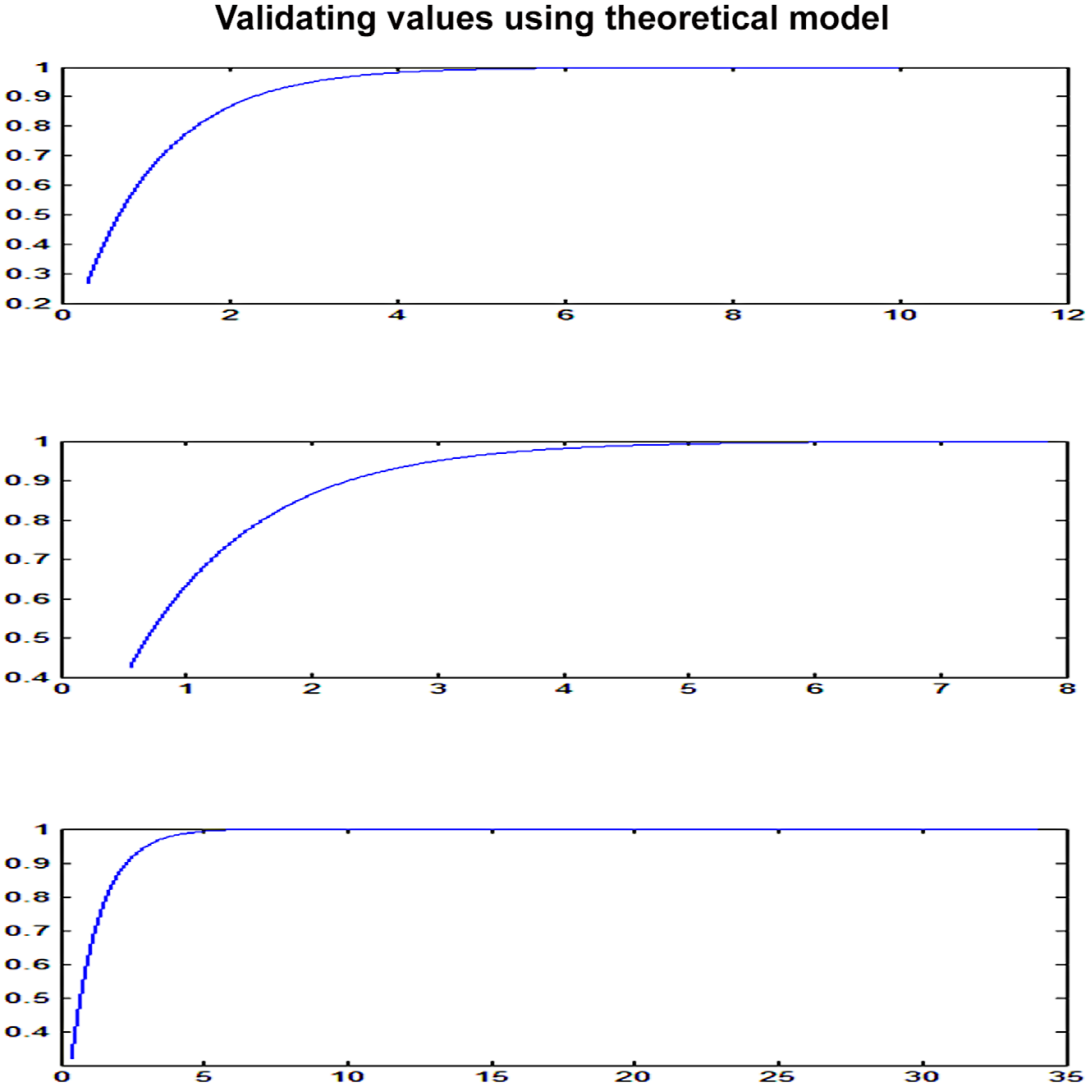
Validating theoretical model-predictive (HBO) - slow growth during therapy – Using Experimental Data from graph 2D.

A theoretical plot of tumor growth progression based upon geometric distribution of cell proliferation in a tumor under normal condition has been shown in Figure 4, 5, 6. We used one of the final equations proposed in a theoretical tumor model [42], [43] based upon our simplifying assumptions of cell proliferation rate with respect to time and initial tumor volume. The equation was solved using MATLAB program. The mathematical model and the program have been given in Appendix S1. Modeling is to verify that the data reflects simplifying assumptions, at least approximately. The key assumptions are that *(a)* The SDs is equal at all times in all groups, (b) The errors are symmetrical, and (c) The growth curve is adequately represented by the proposed parametric form (e.g., a straight line, a quadratic or a spine). We generated data under the assumptions that (a) the true, underlying growth curves are all equal.

**Figure 6:**
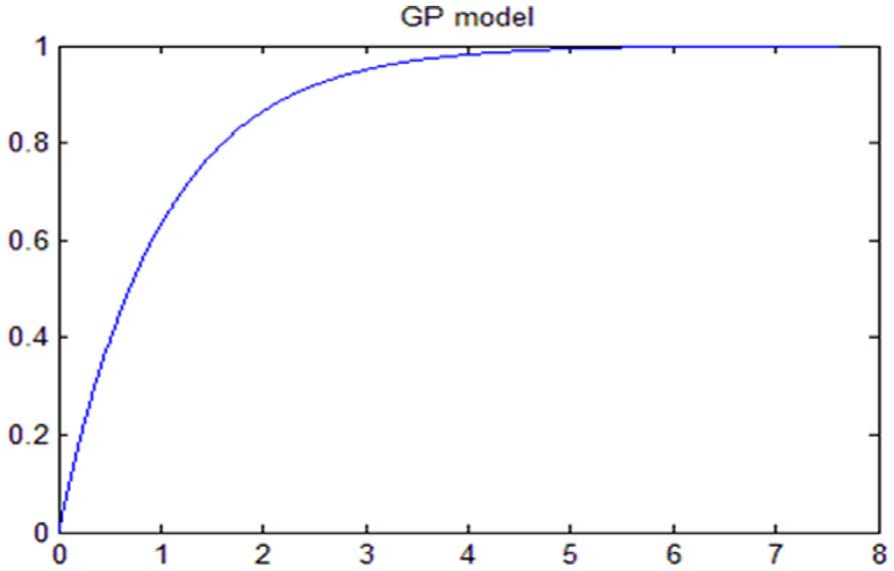
Tumor growth progression (Geometric Progression-Theoretical model).

## Results

A total of 22 tumor bearing mice were studied that includes 7 mice in controls group (normobaric normoxia) and 15 mice in HBO group. (Table 1) Tumor measurements started approximately 5 weeks post tumor inoculation, when the tumors were approximately 1.4 cm^3^.

### Latency Period

The spontaneous tumor cell suspension of density 2.5×10^6^ cells/ml was injected subcutaneously into mammary fat pad. The latency period observed in various set of experiments was found to be approximately 60 days (Figure 1).

### Effect of HBO as Single Modality and Survival

HBO therapy at daily exposure to HBO at 1.1 bar and 1.2 bar for 120 min for 21 days was tolerated well by C3H mice. During the entire period of observation i.e., until they survived, tumor volume increased both in the control (Figure 2A) as well as in the HBO treated group (Figure 2B). In treated group, growth and progression was rapid and at the same time it reached much higher volume, however growth and progression was slow in the control group. They also showed marked difference in growth progression with respect to time in each of the mice basically depicting individual heterogeneity in tumor growth pattern (Figure 2D).

The response of the HBO therapy on tumor progression when administered for 21 days was shown in Table 1 & Figure 2C (i). The differential response of HBO therapy on the tumor progression and metastasis when administered for 7 days and 21 days during and post therapy period has been shown in Table 1 & Figure 2B. The response has been dose and duration dependent, i.e., when the dose is less and duration of administration is short, there is an early relapse or rapid rebound growth and vice versa (Figure 2B, 2C (i)).

We observed slow tumor growth or no tumor enhancing effect during the period of HBO therapy and the effect has been shown in Figure 2C (ii). It clearly showed no cancer enhancing effect which was due to the DNA damage in response to ROS accumulation as explained in the Diagram S1.

Post HBO induces accelerated growth: On long term follow up, we also observed tumorigenic effect post HBO therapy in mice. The HBO treated tumors showed marked rapid tumor progression and a rebound growth post HBO therapy attaining the tumor volume much higher than in the control group at the end survival point (Figure 2D). The rapid tumor progression (high virulence in nature) was observed in animals where the tumor volume on its first appearance was greater (Figure 3). Apart from the tumor growth pattern, we also studied survival period. The survival end point or survival duration between treated (HBO) and untreated group (control) was significantly less but it was not statistically significant (Table 1). The lifespan of HBO treated mice was found to be less {(median value ±2 SD, 91 days(86 D to 95 D)} than control group i.e., tumor induced mice who did not receive therapy {median value ±2 SD, 104 days (52 D to 143 D)} in absolute terms, however, statistically the difference was insignificant (*P  = 0.731).

### Results from the Theoretical Tumor Growth Progression Model

This finding of tumor reaching asymptotic limit was also validated by our own experimental results (Figure 4, 5, 6). The theoretical model based upon natural geometric progression of cell proliferation in a tumor showed a uniform symmetrical tumor growth progression reaching asymptotic limit at a certain period of time under all ideal state conditions. A similar trend was observed when the data (tumor volume with respect to time period) from the experimental study were taken as an input to derive the results using the theoretical model. The results were found to be comparable which validated the model as far the asymptotic limit is concerned (Figure 4, 5, 6). Experimental results however showed marked asymmetry in the tumor growth in each experimental C3H mouse even though all the underlying factors such as person taking daily measurements, maintaining the diet, nutrition, day and night cycle, room temperature etc that may introduce fair amount of error were kept constant. Though the tumor growth progression appears to reach a plateau under both normal and experimental condition similar to results obtained in theoretical model but the reason for the asymmetrical growth progression in each of them with respect to time period (growth rate varied between each mice) could be due to wide host-tumor response or immunogenicity thus limiting the usefulness of C3H mice to be an ideal preclinical cancer therapeutic model.Thus for an effective cancer therapeutic study, we need to use or develop genetically engineered mice for the results to be reproduced in human being.

## Discussion

All normal differentiated cells meet their energy needs by respiration of oxygen relying on mitochondrial oxidative phosphorylation, whereas cancer cells meet their energy needs in great part by fermentation i.e. Warburg effect [31] **.** Tumors require nutrients and oxygen in order to grow. Both oxidative metabolic as well as aerobic glycolysis pathway is involved in cancer cell proliferation and differentiation. Optimizing oxygen therapy is essential in cancer therapeutics. Extrinsically modulating oxygen supply under varying pressure for treating dreadful pathological conditions like cancer is basically aimed at modifying factors involved in cellular metabolism. A better understanding of various phenomena occurring within the tumor microenvironment as a result of various extrinsic & intrinsic influences may provide much needed answer to the complex issue of finding appropriate, safe and effective therapeutic modalities in cancer. We tried to understand the Cellular dynamics i.e., tumor growth in an orthotopic tumor model under normal ambient condition & under stress i.e., oxygen supply at higher Atmospheric Pressure**.** HBO is known to elevate levels of dissolved oxygen (diffusion) raising the concentration of free radicals (ROS) at the level of target cancer cells. It also eliminates tumor hypoxia in regions poorly supplied with oxygen. Tumor microvasculature generally fails to adequately perfuse inner (central) region of the tumor with oxygen making them less sensitive to chemo [32] or radiotherapy due to lack of free radical or oxygen species. HBO helps in diffusing oxygen to these poorly vascularized tumor region making cancer cells more sensitive to radio or chemotherapeutic drugs by promoting ROS activity within the cell. While HBO reduces tumor hypoxia, it may also reduce net tumor micro vascular blood flow. High external pressure exerted on the arterioles or capillaries results in reduction in tumor microcirculation, compromising oxygen perfusion as well as micronutrient supply to the dividing cell. Thus net change in the blood flow volume, oxygen perfusion & diffusion during and after HBO therapy is open to scrutiny and may vary with level & the duration of external pressure being exerted on the vessels and the site of intervention [12]. We documented blood flow alteration as a result of change in vascular architecture, viscosity, elasticity, autonomic regulations, pressure modulation on the sprouting capillaries and arterioles both in theoretical and actual experimental conditions [11],[13]. Thus, different protocol developed by different workers with respect to pressure gradient and duration of HBO exposure probably led to wide variation in the results and controversies surrounding HBO therapy in cancer. Keeping in mind the above mentioned issues and our past experience of working in the field of physiological fluid dynamics, we designed the present research work i.e., to re-evaluate the effect of HBO exposure (stress) on the tumor growth, progression and length of survival in a pre-clinical cancer therapeutic small animal C3H mice model. The basis of subjecting mice to HBO was to modulate oxygen concentration and eliminate hypoxic region, increase sensitivity of chemo or radiotherapy and understand underlying regulatory pathways, cellular energetic and tumor microenvironment. The study included daily exposure of C3H mice at hyperbaric (1.1 bar, 1.2 bar) hyperoxia for 120 min for 21 days in a hyperbaric chamber. HBO is the administration of (100%) O_2_ at higher atmospheric pressure. With 4 atmospheres absolute of oxygen, a 12 fold rise in tumor P0_2_ occurred with a 15–50 fold increase in the pO_2_ of normal tissues [33]. Some investigations have studied direct tissue polarography which indicates that external HBO effectively increases oxygen levels [34]. As reported in past studies, we also did not observe any cancer enhancing effect during the period of HBO therapy. This inhibitory effect could be due to the cytotoxic effect or enhanced DNA damage, enhanced apoptosis and delayed DNA repair due to exposure to free oxygen radicals [16],[22]. Free oxygen radicals also have mutagenic effect on the proliferating cells [20]. The higher atmospheric pressure exerted on the walls of small arterioles and capillaries reduces oxygen tissue perfusion, raises the mean intra-arterial pressure effectively reducing the net blood flow & nutrients supply. Reduced blood flow and nutrient supply during the phase of therapy may further suppress the tumor growth. Improved tissue oxygen diffusion of the hypoxic region during HBO therapy may also down regulates the process of angiogenesis thereby preventing acceleration of tumor growth [35]. However controversy exists, it is also reported that HBO have tumor promoting effect. There are several lines of evidences supporting this hypothesis [23]. It influences wound healing through attenuating apoptosis and decreasing inflammation [36] and may also promotes vasculogenesis [37]. Interestingly, on long term follow up, we also observed tumorigenic effect post HBO therapy in mice. A similar finding was also reported in a study conducted in human participants suffering from head and neck cancer and treated with HBO [8]. Impaired DNA repair mechanism, modulation of blood supply & nutrients, mutagenic effect on proliferating cells & aneuploidy may possibly play a role in promoting uncontrolled cell proliferation leading to rebound tumor growth. Any growing normal tissues or tumors require nutrients and oxygen in order to grow. Improved blood flow & nutrient supply and oxygen perfusion following cessation of hyperbaric pressure (high external hydrostatic pressure) may promote rapid tissue repair and wound healing. Aneuploidy linked to mutagenic action of HBO on dividing cells may also lead to an abnormal rapid proliferation and delayed DNA repair [23], [25], [38]-[40]. Thus, based upon our as well as other workers finding, we believe that HBO indeed has dual effect on tumor growth i.e., tumor growth retardation followed by acceleration during and after HBO therapy respectively. The cell-cell interactions, cell proliferation and tissue function depends upon their microenvironment and we have been studying the role of such modulators and other physical influences for the last many years [11]-[13], [41]. The dual effect of HBO on the tumor growth progression during and after cessation can be explained & elaborated on the basis of its having action on multiple targets i.e., on cell proliferation, DNA damage & repair mechanism, mutation & aneuploidy, and blood flow dynamics in Diagram S1 & S2. We found tumor volume and dose dependent effect of HBO on tumor progression. The dual mechanism of HBO therapy on the blood flow dynamics i.e., increasing oxygen tissue diffusion and reducing oxygen/nutrient perfusion may be responsible for its dose dependent response on the tumor growth & progression. In vitro studies on cultured mammalian have showed mutagenic effect of increased HBO exposure (4 ATA). A dose-related induction of chromosome damage was measured in V79 cells with the MNT with increasing exposure time (0.5–3 h). The clastogenic (chromosome-breaking) effect of HBO in V79 cells correlated very well with the increase in DNA damage obtained with the comet assay in the same cell population [20].

### HBO and Survival

The lifespan of HBO treated mice was found to be less {(median value ±2 SD, 91 days(86 D to 95 D)} than control group i.e., tumor induced mice who did not receive therapy {median value ±2 SD, 104 days (52 D to 143 D)} in absolute terms, however, statistically the difference was insignificant. As HBO therapy achieve desired tumor suppression, it may also have some negative systemic effect of oxygen toxicity. The detrimental effect of exposure at high concentration is due to the reactive oxygen species (ROS). The systemic toxicity, accelerated tumor growth post HBO therapy and evident lung metastasis in gross findings may be some of the responsible factors for shorter life span amongst the treated group. The reversal of reduced blood flow or improved blood flow following cessation of HBO therapy may be another responsible factor for enhanced tumor growth owing to improved nutrition and oxygen perfusion as well as transportation of tumor cells to distant location causing metastasis.

The above mentioned negative effect on survival duration is also substantiated by a study, which showed improved survival when mice were subjected to low oxidative stress created with moderate exercise regime and not high oxidative stress that is generally created with HBO exposure [42].

### Asymmetrical Growth and Theoretical Model

Theoretical model [43], [44] predicted tumor growth reaching asymptotic limit at a certain period of time (Appendix S1). This finding of tumor reaching asymptotic limit was also validated by our own experimental results (Figure 4, 5, 6). Theoretical model based on geometric progression of cell proliferation showed symmetrical growth pattern which was as per our initial assumptions to consider C3H mice as homogenous tumor model. On the contrary, we observed marked asymmetry in the tumor growth in each of the experimental C3H mouse which may limit the utility of spontaneous cancer model as an ideal pre-clinical therapeutic model. The differential growth pattern needs to be reviewed and there is also a need to search an alternative small animal model. We probably need an animal model such as genetically engineered mice or some other specific strain which showed symmetrical tumor growth progression for the results to be reproduced in human being.

## Conclusion

We did not find any cancer enhancing effect during the HBO therapy. However, acceleration in tumor growth was observed following completion of HBO therapy. At the same time, mice subjected to HBO therapy lived shorter as compared to those not exposed to HBO therapy (survival period from induction of tumor cells until deaths). The effect of HBO in terms of causing DNA damage due to exposure of proliferating cells to free radicals (ROS) as well as compensating tumor hypoxia thus making cancer cells more chemo or radio sensitive is well studied. The mutagenic effect of HBO exposure on proliferating cells causing aneuploidy and the mechanism of its action on tumor vascular remodeling and micro-environment has not been well studied. There is also a need to understand DNA damage and repair pathways. Based upon the preliminary results obtained in our study, we tried to explain underlying pathophysiological mechanisms that may be linked to HBO therapy (Figure 1 & 2) in terms of its dual effect. Though our experimental results validated a well established theoretical model on cancer cell proliferation rate but wide degree of individual variations in tumor growth progression suggested a need for an alternative approach in pre therapeutic cancer modeling so that the results can be reproduced in human being.

## Supporting Information

Diagram S1 **Showing the study plan and the effect of HBO therapy.**
(TIF)

Diagram S2 **Tumor microenvironment showing micro vascular modulation.**
(TIF)

Appendix S1 (TIF)
