# Supplementary figures and images for: Re-Evaluate the Effect of Hyperbaric Oxygen Therapy in Cancer - A Preclinical Therapeutic Small Animal Model Study
Source: PLoS One. 2012 Nov 7;7(11):e48432. doi: 10.1371/journal.pone.0048432 (PMC3492351; doi:10.1371/journal.pone.0048432)

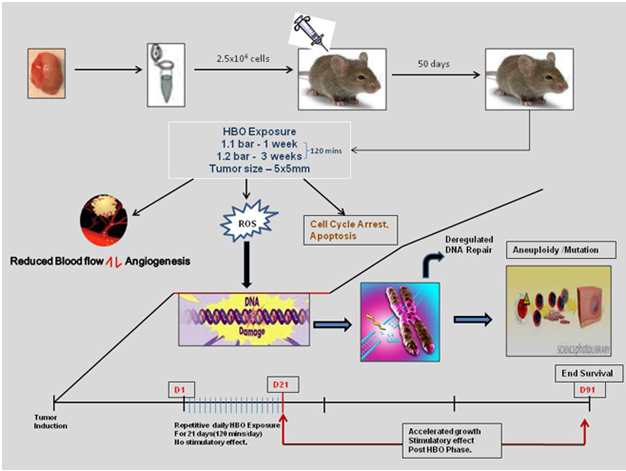

Supplement: Diagram S1 — Showing the study plan and the effect of HBO therapy. (TIF) [file pone.0048432.s001.tif]

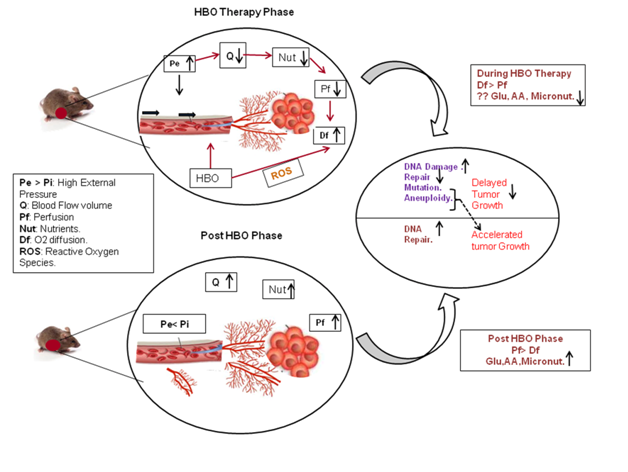

Supplement: Diagram S2 — Tumor microenvironment showing micro vascular modulation. (TIF) [file pone.0048432.s002.tif]

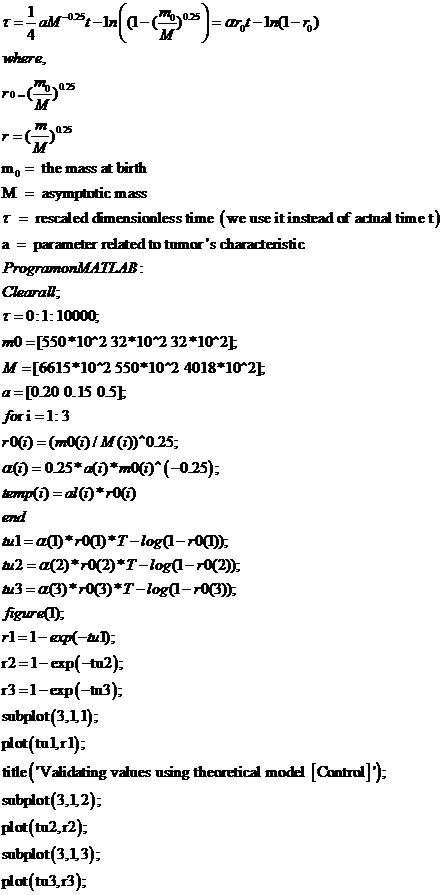

Supplement: Appendix S1 — (TIF) [file pone.0048432.s003.tif]
